# Chronic Stress Disturbs Metabolome of Blood Plasma and Urine in Diabetic Rats

**DOI:** 10.3389/fpsyt.2018.00525

**Published:** 2018-10-23

**Authors:** Yu Ren, Cheng-hua Yang, Zhu-man Li, Zhen Yang, Zhi-jun Xiao, Jing-jing Duan, Ting Zhou, Feng Xu

**Affiliations:** ^1^Fengxian Hospital, Southern Medical University, Shanghai, China; ^2^Fengxian Hospital, Jinzhou Medical University Graduate Base, Shanghai, China

**Keywords:** chronic unpredictable mild stress, depression, diabetes, metabolome, rats

## Abstract

About 30% of diabetes patients suffer from varying degrees of depression. Diabetes itself is associated with abnormal carbohydrate and energy metabolism. Whether chronic stress-induced depression-like behavior impacts the metabolome of blood plasma and urine in diabetes is not clear. This study aimed to investigate the effect of chronic stress on metabolome of plasma and urine in spontaneously diabetic Goto-Kakizaki (GK) rats. The GK rats were subjected to 8 weeks' chronic unpredictable mild stress (CUMS) to induce depression-like behavior. Metabolome analysis of blood plasma and urine using liquid chromatography mass spectrometry (LC/MS) was performed. Multivariate data analysis was used to evaluate the data. Behavior and biochemical assay confirmed the successful establishment of CUMS-induced depression-like behavior model in rats. Disturbance of 20 plasma metabolites and 16 urine metabolites were altered in CUMS-induced depression GK rats as compared to control ones. These disturbed metabolites were involved in fatty acid metabolism, sphingolipid metabolism, phenylalanine metabolism, citrate cycle, glycolysis, glutathione metabolism, and nicotinate and nicotinamide metabolism. This study suggest that chronic stress-induced depression-like behavior may further disturb diabetes-itself energy metabolome. The plasma and urine lipid metabolites monitoring may be useful for early detection of depression in patients with diabetes mellitus.

## Introduction

Lifelong medication, risk of various complications, and heavy care burdens result in psychosocial stress in patients with diabetes mellitus, which is detrimental to mental health and often lead to depression (1). About 30% of diabetes patients experience varying degree of depression (2, 3), and therefore have worse outcomes (4, 5). The pathological mechanisms underlying depression are complex and still not fully understood. Abnormity of catecholamine neurotransmitters in the brain have been considered the primary cause of depression, Levels of 5-hydroxytryptamine (5-HT), noradrenaline (NE), and dopamine (DA) are generally reduced in patients with depression (6). Neuroinflammation is also associated with the occurrence and development of depression (7). Recently, evidences suggested that depression might be related to carbohydrate and energy metabolism abnormity (8, 9).

Since diabetes mellitus itself is a chronic metabolic disease (10), depression co-morbid is speculated to affect the abnormal metabolome in diabetics, and to increase cardiovascular complication risk (11, 12). Accordingly, early diagnosis of depression comorbidity is of importance in diabetes management. However, early identification of depression is difficult in clinical practice due to its complex etiology (13).

Metabolomics is a top-down systems biology approach for studying the metabolic responses of living systems to genetic or environmental stimuli (14, 15). It has been increasingly used to diagnose diseases, in pharmacology/toxicology studies, and for the identification of biomarkers (16). Combined metabolomics and proteomics analysis was conducted to identify potential biomarkers in an animal model of depression-like behavior (17, 18).

Among the analytical platforms of metabolomics, LC-MS is recognized as one of the best powerful tools in sensitivity, selectivity and reproducibility (19, 20). Meanwhile, LC-MS has increased the number of lipid classes that can be analyzed, it can separation and identification trace components of complex mixtures (21). So now LC-MS has become a mainstay of metabolomics for metabolite identification.

To better understand the complex metabolome changes that occur in diabetes with depression comorbidity, plasma and urine metabolome was investigated in spontaneous diabetic GK rats with depression-like behavior. This study aimed to provide a method for early identification of depression in diabetes based on changes in metabolites in plasma and urine.

## Materials and methods

### Animals

Five weeks old male spontaneous diabetic GK rats were purchased from Shanghai SLAC Laboratory Animal Co., Ltd. (Animal Quality Certificate: 2007000562918). The rats were housed in the Laboratory Animal Center, East China Normal University, Shanghai (Animal Experiment License: SYXK 2010-0094) for 7 weeks in a SPF-grade lab until they developed diabetes (with postprandial blood glucose level ≥ 11 mmol/L). Then they were randomly divided into the normal control group and CUMS model group (12 rats per group). Animal procedures were performed in accordance with the guidelines of the Institutional Ethics Committee.

### Chemicals

LC/MS-grade acetonitrile and HPLC-grade methanol were purchased from Merck (Darmstadt, Germany). Methanoic acid was purchased from CNW Company (Germany). All other chemicals were of analytical grade and were purchased from Sigma (St. Louis, MO, USA). Leucine-enkephalin (LE) standard was obtained from Sigma Corporation (St. Louis, MO, USA). Watson's distilled water was used.

### Behavior testing–open-field test

The open-field test was conducted in a quiet room (22). Briefly, the Open-field consisted of an opaque plastic box (100 × 100 × 40 cm) divided equally into 33 × 33 cm^2^ squares. Rats were placed in the center of this field, and the number of squares rats moved and standing times were monitored using a ZS-ZFT Video Analysis System (ZSDC Science and technology Co., Ltd., China) as indexes of locomotion activity and exploratory behavior, respectively. Animals with abnormal behavior scores >120 or < 30 were removed from the experiment prior to chronic stress. 10 rats in control group and 11 in model group ultimately completed the experiment. The test lasted 5 min and was conducted before and after 8 weeks of chronic stress.

### CUMS-induced depression-like behavior model

The CUMS-induced depression-like behavior model was established in GK rats (23, 24). Rats in the CUMS model group were individually housed in cages for 8 weeks with food and water *ad libitum*. Each rat was exposed to the following stressors in random order: restraint (activity restriction in a bottle, 1 h), hot water swimming (45°C, 5 min), cold water swimming (4°C, 5 min), clip tail (1cm from the end of the tail, 1min), cage tilting (45°, 24h), horizontal shaking (10 min), damp padding (24 h), noise interference (10 min), and day/night inversion (24 h). The same stress was not used twice continuously in a row to avoid prediction. After using 6–7 times of each stress treatment randomly, the rats were relocated to their cages. Control rats were fed normally without any stress during this period.

### Biochemical assay

Before and after model establishment, blood was collected from eye can thus, kept in heparinized tubes, and centrifuged at 3,000 × g at 4°C for 5 min to obtain plasma. Plasma serotonin and dopamine levels were measured by enzyme-linked immunosorbent assay (CUSABIO, USA) according to instruction.

### Metabolome analysis using LC/MS

After model establishment, blood was collected as above, and the supernatant plasma was stored at −80°C. Rats were placed in metabolism cages and urine samples were collected every 24 h, aliquoted and stored at −80°C. All samples were then thawed for 15 min and vortexed for 5s prior to analysis. A total of 100 μL of plasma/urine sample was mixed with 300 μL of methanol, vortexed for 30 s, and left undisturbed for 20 min. After centrifugation at 12,000 × g for 15 min at 4°C, 200 μL supernatant was extracted for LC/MS assay.

#### LC/MS analysis

LC/MS (Agilent, 1290 Infinity LC, 6530 UHD and Accurate-Mass Q-TOF/MS) were used to analyze the samples. The chromatographic separation conditions are as follows. Each sample (4 μL) was injected into a C_18_ column (Agilent, USA, 100 × 2.1 mm, 1.8 μm) at 4°C, and the following mobile phases were used at a flow-rate of 0.4 mL/min at 40°C: H_2_O with 0.1% methane acid (A) and acetonitrile with 0.1% methane acid (B). Gradient elution values are shown in Table 1. Mass spectrometry analysis was performed in V flight tube detection mode with nitrogen as the atomization cone gas in both positive and negative ion modes. The source temperature was set at 100°C, the extraction cone was at 4v, and a cone gas flow of 50 L/h was used in both modes. Capillary voltage was set at 4.0 and 3.5 kV, the sampling cone was 35 and 50 kV, the desolvation temperature was 350°C and 300°C, respectively, and the desolvation gas flow was set at 600 and 700 L/h, respectively. Mass spectrometric data was collected in centroid mode from 50 to 1,200 m/z.

**Table 1:** Mobile phase gradient.

**Time (min)**	**Flow (mL/min)**	**Pressure limit (bar)**	**Solv ratio B (%)**
0	0.35	800	5
1	0.35	800	5
6	0.35	800	20
9	0.35	800	50
13	0.35	800	95
15	0.35	800	95

The peak height for the internal standard was continuously monitored during analysis to ensure signal stability. Quality control (QC) procedures were used to validate the methods and to ensure stability. 50 μL QC samples prepared by pooling identical volumes of individual plasma sample. To ensure that the system was suitable for use, 5 pooled QC samples were run prior to analysis in each ion mode. Five ions (min_m/z) were selected to evaluate the relative standard deviation (RSD) of retention time, m/z, and peak area. The peak area RSD values of reproducibility for most of the compounds were <10%.

### Identification of metabolites

OPLS-DA and variable importance for projection statistics (VIP > 1) were used to select significant variables driving group separation. Metabolites were represented by chromatographic peaks in total ion current chromatograms (TICs), and identified by comparing their chromatographic retention times and MS spectra with the available references and the METLIN database. In addition, the Kyoto encyclopedia of genes and genomes (KEGG, http://www.genome.jp/kegg/) biochemical database was used to interpret possible pathways involving the identified metabolites.

### Data processing and statistical analysis

Data from the behavioral and 5-HT/DA level tests are expressed as means ± standard errors of the mean. Student's *t*-tests with two tails and unequal variance were performed with GraphPad software; significant differences were indicated by *p* < 0.05. Mass Profile software was used for peak extraction, retention time (RT) alignment, peak alignment, and deconvolution analysis. Finally, data were imported into SIMCA-P software (v13.0, Umetric, Umea, Sweden) for PCA, PLS-DA, and OPLS-DA. VIP > 1 and *p* < 0.05 were considered statistically significant.

## Results

### Behavioral testing-open-field test

An open-field behavioral test revealed significant differences between 2 groups after 8 weeks of chronic stress treatment. At the end of 8 weeks CUMS, the locomotion and exploratory scores are decreased in CUMS model group rats compared to baseline (14.1 vs. 37.4, *p* < 0.01, and 7.6 vs. 14.2, *p* < 0.01, respectively). No different change occurred in control group rats (Table 2).

**Table 2:** Open-field test before and after model establishment.

**Behavioral**	**CUMS group**		**Control group**	
	**Before CUMS**	**After CUMS**	**Before CUMS**	**After CUMS**
Locomotion scores	37.40 ± 9.44	14.10 ± 3.00^**^	37.90 ± 7.97	39.80 ± 9.24
Exploratory scores	14.10 ± 2.38	7.60 ± 2.17^**^	15.20 ± 4.26	14.10 ± 2.33

*Significant difference within depression group rats before vs. after model establishment*,

** *P < 0.01*.

### Biochemical assay

Biochemical test was done to show that chronic stress treatment also reduces serotonin (5-HT) and dopamine (DA) levels in CUMS model group rats [from 62.09 to 22.17 ng/mL (p < 0.01) and from 0.55 to 0.21 ng/mL (*p* < 0.01), respectively]. Neither serotonin nor dopamine levels change over time in normal control group rats (Table 3). These results confirm that CUMS successfully induce depression-like behavior in GK rats.

**Table 3:** Biochemical tests before and after model establishment (ng/mL).

**Biochemical tests**	**CUMS group**		**Control group**	
	**Before CUMS**	**After CUMS**	**Before CUMS**	**After CUMS**
Serotonin levels	62.09 ± 12.76	22.17 ± 4.28^**^	61.71 ± 12.94	58.31 ± 10.01
DA levels	0.55 ± 0.07	0.21 ± 0.05^**^	0.55 ± 0.13	0.50 ± 0.12

*Significant difference within depression group rats before vs. after model establishment*,

** *P < 0.01*.

### Metabolome analysis using LC/MS

**Figure 1:**
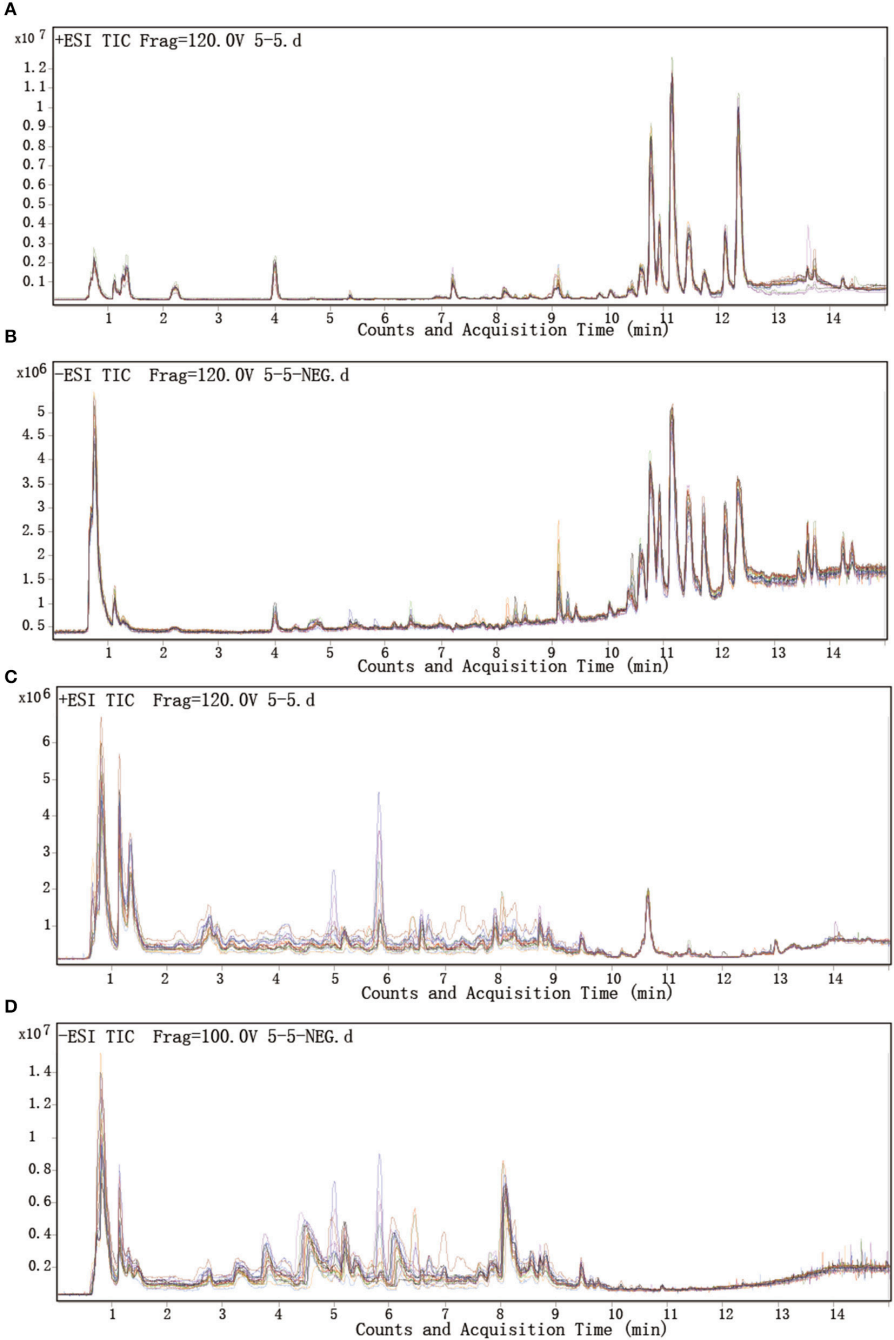
Representative LC-Q/TOF-MS analysis of total ion chromatograms for plasma and urine. **(A,B)** Plasma samples in positive and negative mode. **(C,D)** Urine samples in positive and negative mode.

**Figure 2:**
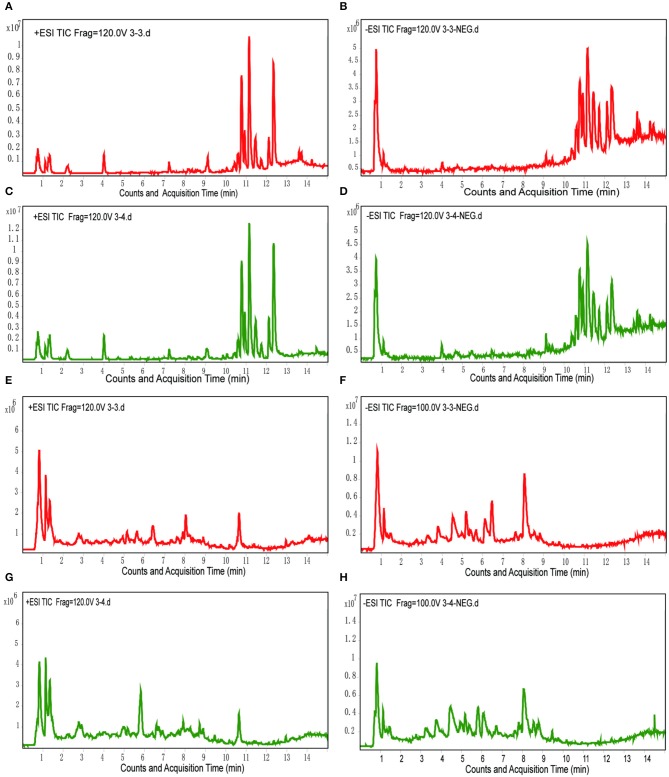
Typical LC/MS TICs of plasma and urine samples from the two groups. **(A,B)** Plasma samples from the model group in positive and negative mode. **(C,D)** Plasma samples from the control group in positive and negative mode. **(E,F)** Urine samples from the model group in positive and negative mode. **(G,H)** Urine samples from the control group in positive and negative mode.

Total ion chromatograms (TICs) were visually inspected to examine the stability and reproducibility of the instrument. The results indicate that it was functioning optimally (Figures 1A–D). Typical LC/MS TICs for plasma and urine samples from the two groups (Figures 2A–H) indicate that the analysis was highly reproducible. Quality control tests of LC/MS TICs were performed for plasma and urine samples. As shown in Table 4, RSD values for the quality control samples were all <10%, indicate that the analysis system was stable.

**Figure 3:**
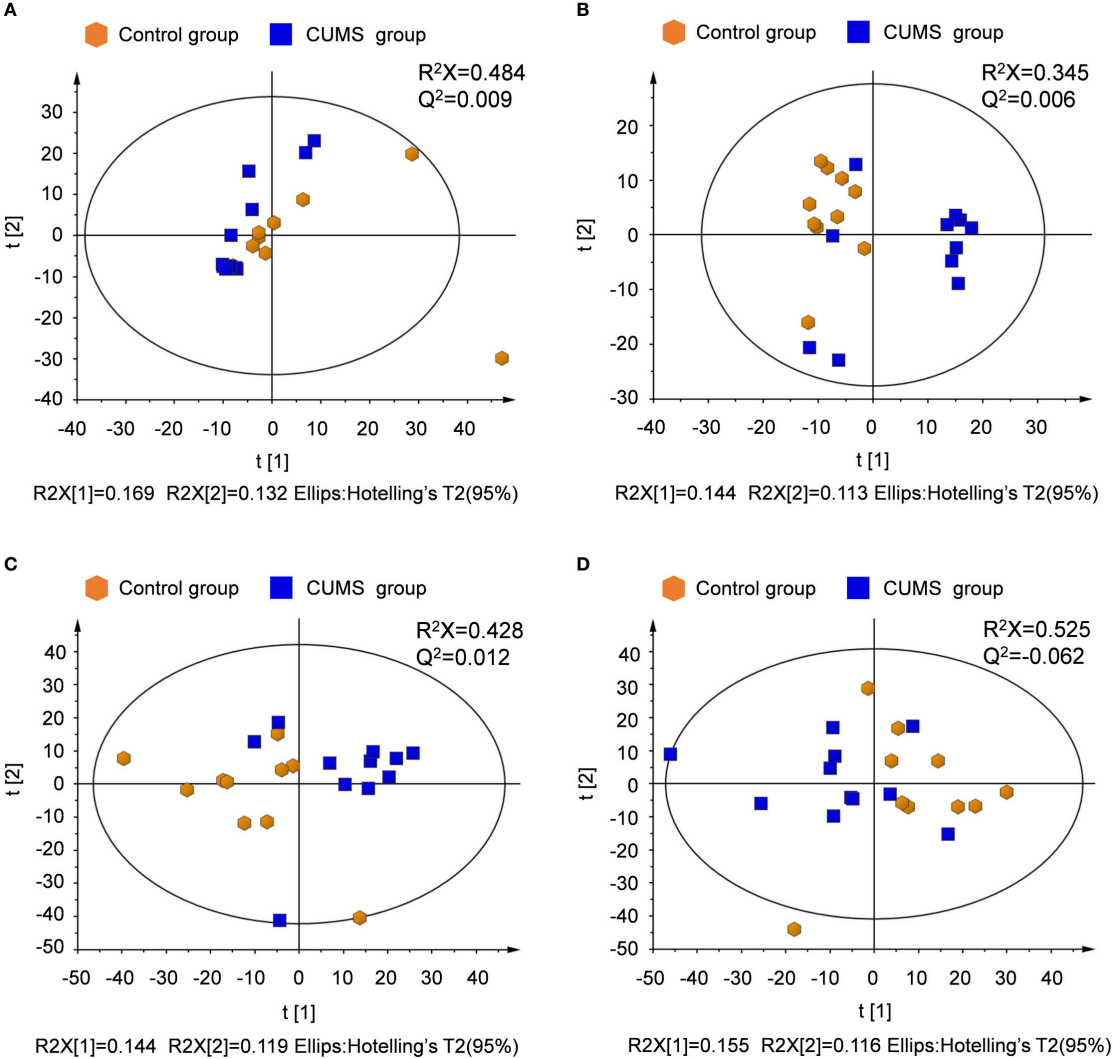
PCA score plots derived from LC-MS analysis of plasma and urine samples from model and control group rats. **(A)** Plasma in positive ion mode, **(B)** plasma in negative ion mode, **(C)** urine in positive ion mode, **(D)** urine in negative ion mode.

Unsupervised principal component analysis (PCA) was performed to provide an overview of the LC-MS data. PCA score plots are shown in Figure 3. Significant differences are observed between the normal control and CUMS model groups in both ion modes for plasma and urine samples.

**Table 4:** Method stability for LC/MS of plasma/urine.

**Sample**	**tR_m/z**	**System stability (RSD, %)**	**Sample stability (RSD, %)**
Plasma	8.74_406.3	2.75	4.46
	9.90_307.2	3.11	5.32
	8.79_288.3	3.74	6.54
	9.12_373.3	4.07	5.76
	10.13_129.1	4.25	3.78
Urine	5.04_396.2	3.66	4.34
	6.58_264.1	4.00	5.41
	9.81_269.1	3.22	4.79
	9.13_202.1	4.21	6.56
	7.10_539.2	4.51	6.44

**Figure 4:**
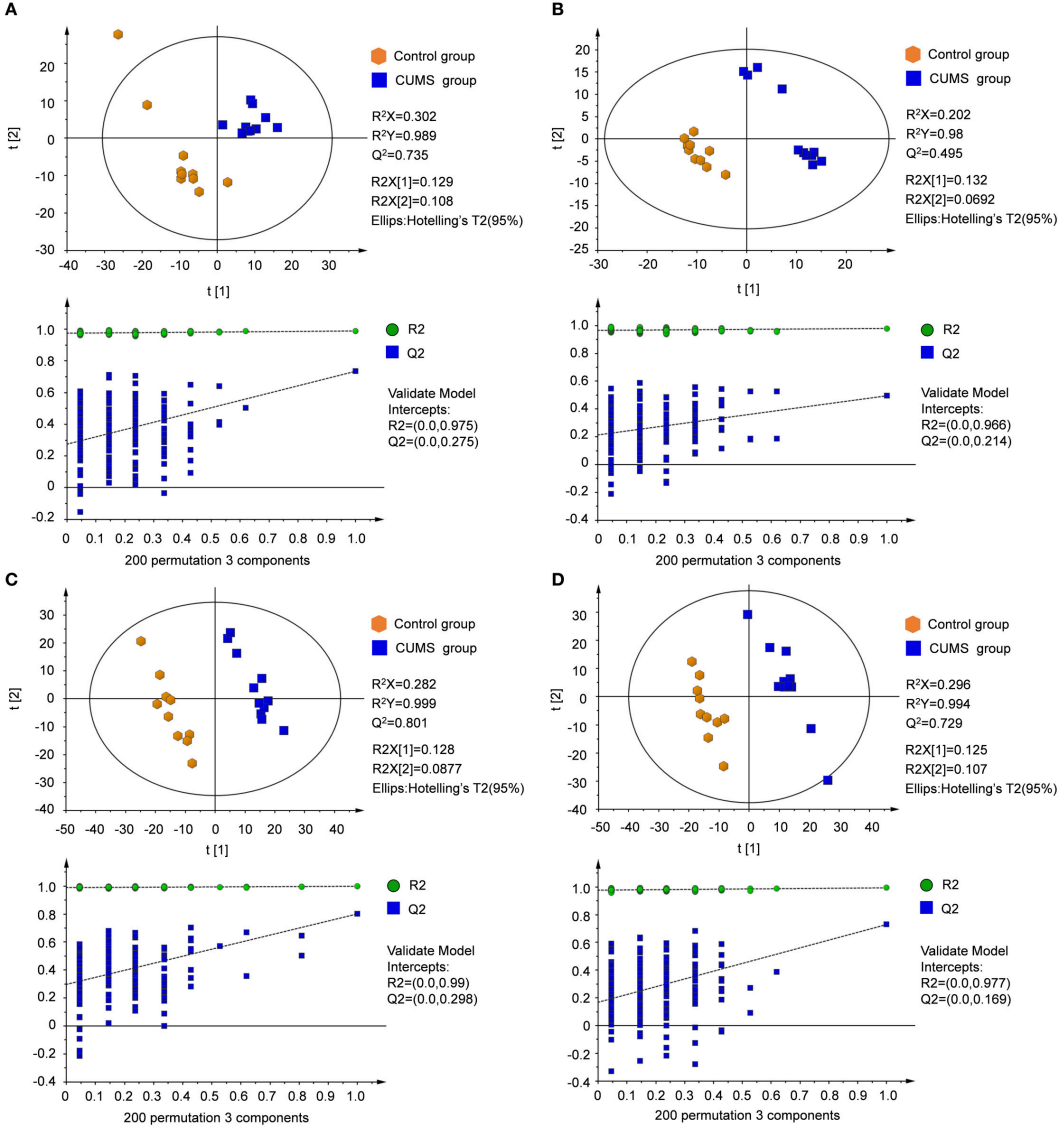
PLS-DA score plots and sorting validation figures derived from LC-MS analysis of plasma and urine samples from model and control groups in different ion modes. **(A)** PLS-DA score plot and sorting validation of plasma in ESI+, **(B)** PLS-DA score plot and sorting validation of plasma in ESI–, **(C)** PLS-DA score plot and sorting validation of urine in ESI+, **(D)** PLS-DA score plot and sorting validation of urine in ESI–.

Supervised partial least squares-discriminate analysis (PLS-DA) was used to examine metabolic patterns and to detect changes in the concentration of individual metabolites in the model. Measures of the quality of the resulting discrimination, including the values of R^2^X, R^2^Y, and Q^2^, are shown in Figure 4. The values of R^2^ and Q^2^ were > 0.5, indicating good fitness and prediction.

**Figure 5:**
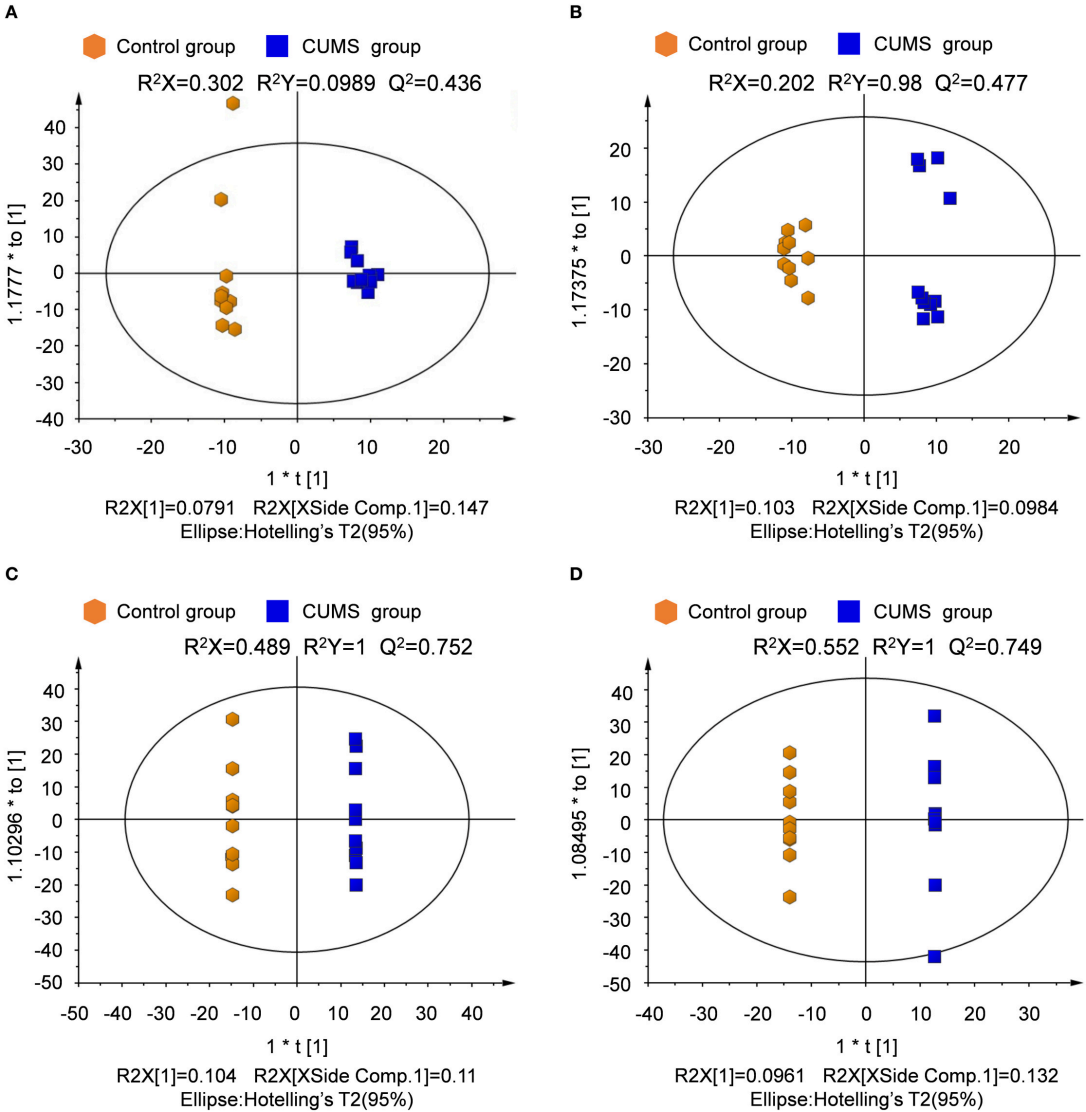
Score plot derived from OPLS-DA model classification. **(A)** Plasma in positive ion mode, **(B)** plasma in negative ion mode, **(C)** urine in positive ion mode, **(D)** urine in negative ion mode.

The orthogonal partial least squares-discriminate analysis (OPLS-DA) model was constructed after the PLS-DA analysis. The key model parameters are summarized in Figure 5. Results for different groups were visualized as score plots to show group clusters. S-plots were used to identify variables that contributed to the classification. Good separation between the groups was observed.

### Chronic stress disturbs metabolome

Differentially-expressed metabolites were identified based on the variable VIP with a threshold of 1.0 from the OPLS-DA model and *p*-value < 0.05 in the *t*-test. Metabolites were identified based on mass assignment and Rt-m/z and were then compared with authentic standards or database resources, such as KEGG (http://www.genome.jp/kegg/) and METLIN (http://metlin.scripps.edu).

**Figure 6:**
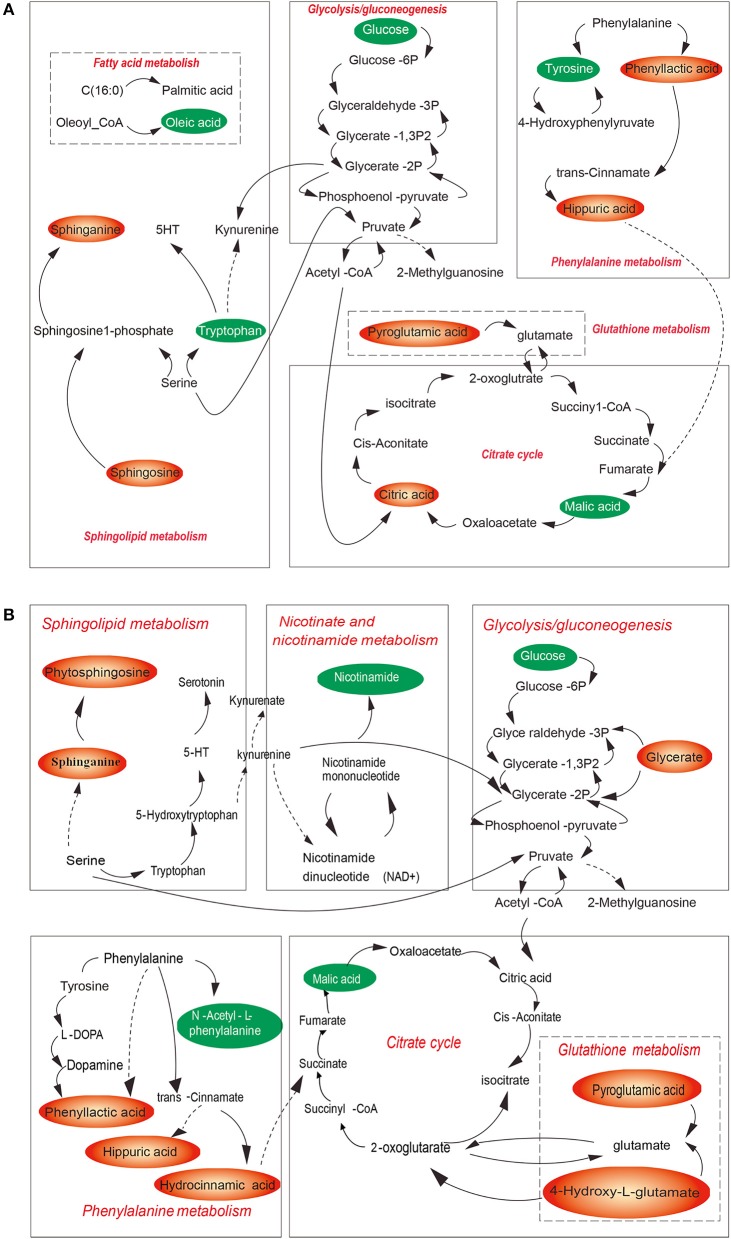
Overview of the metabolic pathways associated with CUMS-induced depression for plasma **(A)** and of urine **(B)** samples. Red metabolites were increased in the model group, green metabolites were decreased in model group. Red pathways are associated with CUMS-induced depression like bebavior.

Among thousands of metabolites, 20 molecules in plasma and 16 in urine were significantly correlated with CUMS-induced depression-like behavior. Specifically, in the plasma of CUMS group, levels of 2-methylhippuric acid, bilirubin, sphinganine, and sphingosine were increased, meanwhile levels of oleamide, oleic acid, L-tryptophan, L-tyrosine, lysophosphatidylcholine (lysoPC), lysophosphatidylethanolamine (lysoPE), and α-D-glucose were decreased. In the urine, 2-methylhippuric acid, hydrocinnamic acid, phenyllactic, 4-hydroxy-L-glutamic acid, pyroglutamic acid, and cholic acid were significantly up-regulated., whereas the levels of oleamide, cortisone, cytosine, hypoxanthine, malic acid, and α-linolenic acid were down-regulated. The plasma and urinary metabolites responsible for the observed difference between CUMS and normal control rats were summarized in Table 5, together with retention time, mass-to-charge ratios, possible pathways, and KEGG IDs (obtained using the KEGG pathway database). These data suggest that changes occurred in the following 7 metabolic pathways in CUMS group (Figures 6A,B): fatty acid metabolism, sphingolipid metabolism, phenylalanine metabolism, citrate cycle, glycolysis, glutathione metabolism, nicotinate and nicotinamide metabolism.

**Table 5:** Metabolites in plasma/urine samples that differed between the control and CUMS-induced depression groups.

**Sample**	**Metabolites**	**VIP**	**RT (min)**	**Mass**	**Trend**	**Fold change (B/A)**	**Metabolic pathway**	**KEGG ID**
Plasma	Bilirubin^+^	2.28	7.96	584.26	↑^**^	0.66	Porphyrin and chlorophyll metabolism	C00486
	Citric acid^+/−^	1.72	1.12	192.03	↑^*^	0.98	Citrate cycle (TCA cycle)	C00158
	2-Methylhippuric acid^−^	1.49	5.26	193.07	↑^*^	0.73	Phenylalanine metabolism	C01586
	Chenodeoxycholic Acid^−^	1.71	10.45	392.29	↑^*^	2.70	Primary bile acid biosynthesis	C02528
	LysoPC (15:0)^+^	1.60	12.07	481.32	↓^*^	−0.23	Glycerophospholipid metabolism	C04230
	LysoPC (22:5)^+^	1.69	11.35	569.35	↓^*^	−0.31	Glycerophospholipid metabolism	C04230
	LysoPE (16:0)^+^	1.68	10.89	453.29	↓^*^	−0.20	Glycerophospholipid metabolism	
	LysoPE (18:2)^+^	2.23	10.54	477.29	↓^**^	−0.27	Glycerophospholipid metabolism	
	LysoPC (15:0)^−^	1.70	12.30	481.32	↓^*^	−0.98	Glycerophospholipid metabolism	C04230
	LysoPC (17:0)^−^	1.58	12.12	509.35	↓^*^	−0.20	Glycerophospholipid metabolism	C04230
	LysoPE (22:1)^−^	2.13	12.59	535.36	↓^**^	−1.11	Glycerophospholipid metabolism	
	Oleamide^+^	1.65	9.84	281.27	↓^*^	−0.24		C19670
	Sphinganine^+^	1.59	10.07	301.30	↑^**^	0.25	Sphingolipid metabolism	C00836
	Sphingosine^+^	1.61	9.82	299.28	↑^*^	0.77	Sphingolipid metabolism	C00319
	α-Linolenic Acid^+^	1.74	13.14	278.22	↓^*^	−1.02	Fatty acid biosynthesis	
	L-Tryptophan^−^	1.73	4.01	204.09	↓^*^	−0.39	Phenylalanine, tyrosine and tryptophan biosynthesis	C00078
	L-Tyrosine^−^	2.17	1.12	181.07	↓^*^	−0.72	Phenylalanine, tyrosine and tryptophan biosynthesis	C00082
	Oleic Acid^−^	1.47	14.41	282.26	↓^*^	−0.73	Fatty acid biosynthesis	C00712
	α-D-Glucose^−^	1.48	0.73	180.06	↓^*^	−0.59	Glycolysis	C02216
Urine	2-Methylhippuric acid^+^	1.62	5.80	193.07	↑^*^	4.40	Phenylalanine metabolism	C01586
	Hydrocinnamic acid^+^	1.43	5.53	150.07	↑^*^	1.50	Phenylalanine metabolism	C05629
	Phenyllactic acid^−^	2.01	7.84	166.06	↑^**^	2.01	Phenylalanine metabolism	
	4-Hydroxy-L-glutamic acid^+^	1.37	1.33	163.05	↑^*^	1.90	Glutathione metabolism	C03079
	Pyroglutamic acid^+/−^	1.37/1.48	0.84/0.83	129.04	↑^*^	1.28/0.65	Glutathione metabolism	C01879
	Cholic acid^−^	1.64	9.62	408.29	↑^*^	2.64	Primary bile acid biosynthesis	C00695
	L-Carnitine^+^	1.38	0.79	161.11	↓^*^	−1.29	Lysine degradation	C00318
	Oleamide^+^	1.65	12.46	281.27	↓^*^	−1.44	Fatty acid biosynthesis	C19670
	Phytosphingosine^+^	2.51	10.25	317.29	↑^**^	0.56	Sphingolipid metabolism	C12144
	Sphinganine^+^	2.21	10.92	301.30	↑^**^	0.69	Sphingolipid metabolism	C00836
	D-Glyceric acid^+^	2.21	5.83	250.00	↑^**^	2.57	Glycerolipid metabolism/Glycine, serine and threonine metabolism	C00258
	LysoPE (20:4)^−^	1.49	11.82	501.29	↓^*^	−1.25	Glycerophospholipid metabolism	
	Malic acid–	1.45	1.06	134.02	↓^*^	−0.94	Citrate cycle (TCA cycle)	
	Niacin (Nicotinic acid)^−^	2.04	0.99	123.03	↑^**^	1.68	Nicotinate and nicotinamide metabolism	C00253
	Nicotianamine^−^	2.25	9.60	303.15	↓^**^	−2.60	Nicotinate and nicotinamide metabolism	
	α-D-Glucose^−^	1.73	0.78	180.06	↓^**^	−3.55	Glycolysis	C02216

“*+” represents metabolites detected in positive mode, “–” represents metabolites detected in negative mode*.

* *p < 0.05*,

** *p < 0.01 compared to the control group. A: control group, B: CUMS model group. Fold-change (B/A) Value: Logarithm of the ratio of B to A (base 2); “–” represents a trend toward a decrease in the B group compared to the A group*.

## Discussion

Up to now, few researches focused on the impact of chronic stress-induced depression-like behavior on blood plasma and urine metabolome in diabetes patients or in experimental diabetic rats, although metabolome analysis has been proposed for screening for depression (25, 26). In this study, we found that the disturbance in lipid/amino acid/glucose metabolism occurred in diabetes rats with depression comorbidity. We identified 20 plasma metabolites and 16 urine metabolites of GK rats with depression comorbidity. In CUMS model group, we noted with interest that lipid metabolite changes occurred primarily in blood plasma, meanwhile amino acid metabolite changes occurred primarily in urine. The different metabolites identified between plasma and urine due to the different content they have.

In this study we found that lysoPC/lysoPE was decreased in blood plasma in CUMS model rats. PC and PE are considered to be related with normal cognition function and memory (27). Therefore, chronic stress-induced depression-like behavior might be contributed by decrease of lysoPC/lysoPE. Besides, plasma sphingosine and urine phytosphingosine, plasma sphinganine and urine sphinganine were increased in CUMS model rats, which might resulted in ceramide increase. High level of ceramide expression in the brain induces depression (28, 29).

It is worth mentioning that among these altered metabolites, 2-methylhippuric acid was increased meanwhile oleamide was decreased both in blood plasma and in urine, respectivelty, although the biological mechanism is not clear right now. Oleamide is thought to be beneficial to enhance the conductivity of 5-HT receptors and neurons (30), meanwhile 2-methylhippuric acid is thought to be detrimental for it induces nervous system damage *in vivo* and leads to depression (31, 32).

In this study, chronic stress decrease plasma/urine α-D-glucose levels in diabetic rats, which was different from other reports (12, 33). This might be due to different experimental animal model. In this work only male rats were used for study. Gender also influences blood glucose levels in depression patients. Additional research is needed to examine these contradictory results.

The application of LC-MS coupled with PCA makes it possible to show the fact that the biochemical changes of CUMS induced depression-like behavior rats are obviously different from those of the normal control ones, but the mechanism is not clear. Chronic stress was known to disturb of homeostasis, and lead to psychological diseases such as depression. HPA axis with the endpoint release of corticosterone into the circulation plays an important role in mediating the neuroendocrine response to stress (34), so we supposed that the possible mechanism is that changes of metabolic profile resulted from chronic stress via the HPA axis, lead to the metabolic disorders in GK rats.

Clinical practice indicates that glycemic control in diabetes with depression could be improved by anti-depressants co-administration (35). Therefore, diagnosis and interventions as early as possible for depression comorbidity are of importance for successful diabetes management. Blood plasma and urine metabolome detection might be useful for this purpose.

## Conclusion

We conclude that chronic stress-induced depression-like behavior may further disturb diabetes-itself energy metabolome. The plasma and urine lipid metabolites monitor may be useful for early detection of depression in patients with diabetes mellitus.

## Author contributions

TZ and FX conceived and designed the projects. YR, CY, ZL, ZY, ZX, and JD performed the experiments and carried out data analysis. YR and CY drafted the article, TZ and FX finalized the paper and provided suggestions to improve it.

### Conflict of interest statement

The authors declare that the research was conducted in the absence of any commercial or financial relationships that could be construed as a potential conflict of interest.
